# 690. High Mortality and Over-representation of Young Women Amongst People Who Inject Drugs Admitted with Infective Endocarditis in Saskatchewan, Canada from 2013-2018

**DOI:** 10.1093/ofid/ofab466.887

**Published:** 2021-12-04

**Authors:** Anmol Gupta, Sandy Kassir, Savio Nguyen, Stephanie Konrad, Stuart Skinner

**Affiliations:** 1 University of Saskatchewan, Regina, Saskatchewan, Canada; 2 Saskatchewan Health Authority, Regina, Saskatchewan, Canada; 3 Indigenous Services Canada, Regina, Saskatchewan, Canada

## Abstract

**Background:**

The province of Saskatchewan has had the highest rates of HIV and Hepatitis C in Canada for over 10 years, the majority of which is related to People who inject drugs (PWID) and with higher proportion of young women. However, the most severe complications of injection drug use (IDU) are infective endocarditis (IE) and its associated sequelae. While high rates of IE have been noted, no data exists to show the burden of infective endocarditis and its clinical outcomes. Thus, we looked to determine the mortality and impact of IE amongst PWID and also establish the epidemiology while comparing to non-PWID IE.

**Methods:**

This is a retrospective chart review of consecutive adult patients (age > 18) admitted for IE, as defined by Duke’s IE Criteria, at tertiary care hospitals in Regina, the capital city of Saskatchewan, between January 1, 2013 and December 31, 2018. PWID were identified through chart documentation of self-reported IV drug use. Outcomes included 1-year mortality, surgical intervention and referral to addiction services.

**Results:**

Of the total 227 patients in our cohort, 130 (57.3%) were female, and the 1-year mortality was 39.2%. PWID related IE comprised 132 (58.1%) of the cohort. In comparison to non-PWID related IE, PWID were younger (median age 38.0, compared to 68.0 for non-PWID), more likely to be female (RR 2.06; 95% CI [1.44-3.04]; p< 0.001), to suffer right-sided disease (RR 9.14; 95% CI [4.74-15.14]; p< 0.001) and less likely to receive surgical management (RR 0.30; 95% CI [0.27-0.77]; p< 0.001). Surgical management was associated with lower mortality (RR 0.40; 95% CI [0.11-0.65]; p< 0.001). Addiction support and treatment also was protective (RR 0.89; 95% CI [0.34-1.21]; p=0.051).

**Conclusion:**

This cohort study of IE episodes shows for the first time the devastating impact of IDU in Saskatchewan and identifies PWID as having a 39% mortality at 1 year, which coupled with their younger age translates into an enormous years of life lost. Additionally, the over-representation of young women amongst PWID IE is consistent with the higher percentage of young women with HIV and HCV infections, and identifies them as a group that is particularly vulnerable to complications of IDU. Targeted programs for PWID, particularly towards young women at risk are urgently needed.

**Disclosures:**

**All Authors**: No reported disclosures

